# Lessons from insiders: Embracing subjectivity as objectivity in victimology

**DOI:** 10.1177/02697580231179489

**Published:** 2023-06-15

**Authors:** Alexis Marcoux Rouleau

**Affiliations:** Université de Montréal, Canada

**Keywords:** Victimology, epistemology, objectivity, subjectivity, insider research

## Abstract

Due to the prevalence of victimization in society, it is likely that many victimologists have been victimized or will be in their lifetimes. This poses a challenge for the field of victimology as traditional, positivist conceptions of ‘good science’ require researchers to be outsiders relative to populations they study. This paper asks: What are the epistemological and practical implications of victimological research conducted by researchers who have firsthand experiences of victimization? What lessons can be retained by other victimologists and researchers in general? How can these epistemological considerations be applied in practice? To answer these questions, I examine the meanings of insider and outsider status and the implications for objectivity and subjectivity as per positivist and standpoint epistemologies. I present the case of victimologists who have been victimized as well as the advantages and disadvantages of this form of insider research. I deconstruct insider–outsider, subjectivity–objectivity dualisms as they pertain to victimologists, concluding that all victimologists can be subjective whether they are technically insiders or not. In closing, I discuss how all victimologists can embrace their own and their participants’ subjectivity as a resource for objectivity by examining location, emotions and bodies, and ethics throughout the research process.

## Introduction

According to Fattah (2010), victimology should be dispassionate, nonpartisan, and objective; what he refers to as new or applied victimology is bad science. This eminent victimologist argues that clinical victimology and victim advocacy have no place within victimology as science due to close emotional and ideological ties with activism and populism, which may lead to increased penal control of victims who are deemed socially expendable. Fattah’s stance on victimology implies that researchers who have lived experience of victimization and an emotional relationship to the subject matter either have no place in victimological research or are doomed to produce bad science.

Yet due to the prevalence of victimization in society, it is likely that many victimologists have been victimized or will be in their lifetimes. In a context where higher education becomes increasingly accessible to marginalized populations (Kanuha, 2000), more and more researchers may have experienced victimization. Stanko (1992) surveyed members of the American Society of Criminology’s Division on Women and Crime—some of whom were victimologists—as to their experiences of sexual harassment. While in graduate school, 59% experienced sexual comments and 17% felt sexually intimidated by an authority figure, whereas 31% experienced sexual comments or intimidation during fieldwork (*n* = 58 women and 7 men). A more recent study among college students found that 83.3% of criminal justice students at a diverse urban college had experienced direct or indirect victimization (*n* = 371, Eren et al., 2019).

If everyone who has experienced a form of victimization were precluded from conducting research, would there be anyone left to do this work? Indeed, anyone can be victimized at any point in their life. I unpack the following questions in this paper: What are the epistemological implications of research on victimization by researchers who have firsthand experiences as victims? What lessons can be retained by victimologists and researchers in general? How can these epistemological considerations be applied in practice?

Such questions fall within a wider debate: the legitimacy of research conducted by insiders, those who have lived experience of what they study. This paper, thus, tackles the insider–outsider debate within victimological research. By focusing on victimologists who have experienced victimization, wider issues of what objectivity means and the role of subjectivity within research are addressed. To do so, meanings of insider and outsider status and the implications for objectivity and subjectivity are discussed and situated within positivist and standpoint epistemologies, the latter consisting of epistemologies formulated by marginalized groups in academia such as women and Black scholars. The case of victimologists who have been victimized is presented, as well as advantages and disadvantages of this form of insider research. I then deconstruct insider–outsider, subjectivity–objectivity dualisms as pertain to victimologists, concluding on the necessity of utilizing a standpoint definition of objectivity that embraces and draws on subjectivity. In closing, I present a practical guide for researchers who wish to embrace subjectivity as a foundation for objectivity, using examples from victimological research.

## Theoretical framework

In this section, meanings of insider and outsider status and the implications for objectivity and subjectivity are discussed and situated within positivist and standpoint epistemologies.

### The insider–outsider debate

The question of whether researchers should or should not be members of the population they are studying has been debated especially in regard to qualitative research (Dwyer and Buckle, 2009). Ethnographers have in turn been encouraged to ‘go native’^1^ to gain more insights into cultures they are studying, and discouraged from being seduced by these cultures, which would entail losing objectivity (Brannick and Coghlan, 2007; Kanuha, 2000). This debate has been critiqued as leaving little space for those who are ‘already native’, that is, insiders researching their own communities (Kanuha, 2000; Serrant-Green, 2002).

Some strengths and limitations of being an insider or an outsider have been identified. Outsiders have traditionally been framed as objective onlookers; more capable of seeing hidden meanings; able to ask naïve questions due to their detachment from the population under study; fostering trust from participants who would rather discuss personal matters with a ‘professional stranger’ than someone from their social circle. However, outsiders have been criticized as unable to understand nor to properly represent the researched population’s interests; and less sensitive to research fatigue among over researched, small populations (Ashley, 2021; Dwyer and Buckle, 2009; Kanuha, 2000; Serrant-Green, 2002; Zempi, 2016). Insiders have been presented as benefiting from preunderstanding of the subject matter; easier access to their research population and being more apt for rapport-building; more likely to properly represent marginalized groups’ experiences, interests, and voices throughout data analysis and knowledge mobilization; and by extension, as more apt to reduce oppressive and extractive dynamics in research (Acker, 2001; Brannick and Coghlan, 2007; Dwyer and Buckle, 2009; Johnson, 2009; Kanuha, 2000; Serrant-Green, 2002; Zempi, 2016). However, insiders may experience fatigue or burnout; their understanding is not guaranteed; by presuming the researcher understands, participants may omit mentioning important aspects; the researcher may over identify with their participants thus be confused as to their role; all in all, insiders are often assumed to conduct inherently subjective ‘*me*search’ (Brannick and Coghlan, 2007; Dwyer and Buckle, 2009; Johnson, 2009; Kanuha, 2000; Kumar and Cavallaro, 2018; Serrant-Green, 2002; Zempi, 2016).

Yet the core issue to the insider-outsider debate is not one of method—whose social location is valid when conducting research—but the underlying understanding of science. Indeed believing the ‘outsider is best’ and that scholars must be separated from those whom they study to undertake rigorous research is ‘simply a reflection of the idea that reason and emotion must be separated’ (Serrant-Green, 2002: 35 discussing May, 1997). The idea that science should be objective, and that objectivity requires value-neutrality and outsider status, has been cause for debate within social and natural sciences: can scientists truly shed their values, politics, and morals, and is this desirable (Gingras, 2017; Knepper, 2001; Rosenberg, 2018)? I now unpack the meanings of objectivity and subjectivity as well as the implications for insiders and outsiders by examining positivist and standpoint epistemologies.

### Positivist epistemologies: objectivity and subjectivity as diametrically opposed

Positivist epistemologies, also referred to as empiricism or objectivism, view science as tied to prediction and progress (Berthelot, 2012; Comte, 1844). Positivism builds on Enlightenment philosophy such as René Descartes’ work and reacts to medieval inquiry such as alchemy, which did not distinguish theological and philosophical from empirical pursuits of knowledge (Comte, 1844; Durkheim, 1894; Harding, 1986). Indeed Descartes (1637) argued for a methodological skepticism involving the shedding of preconceptions through systematic doubt, so as to build sciences upon certainties. Although discussed in the context of physics and mathematics, positivism was extended to social sciences by early sociologists such as Comte and Durkheim (Beauchamp, 2016; Kincaid, 2016). Comte (1844) argued that positivist science is distinct from theology and philosophy because relationships between phenomena are deduced based on the observation of reality rather than through pure imagination. Seeing empirical proof and establishing invariable and universal relationships between phenomena would then allow scientists to formulate rational predictions (Comte, 1844). As for Durkheim (1894), he urged sociologists to practice Descartes’ methodological skepticism to maintain a strict divestment from prenotions, ideology, values, and morals when investigating what he referred to as social facts.

Positivism rests on a series of hierarchical dualisms (Dixon, 1977; Durkheim, 1894; Haraway, 1988; Harding, 1986; Hill Collins, 1986; Watts, 2013) which are summarized in Table 1. Such rigid dichotomies stem from Descartes’ (1637) epistemological-ontological divide, in which knowing and being—and by extension mind and body—are distinct (Harding, 1986; Mills, 1988; Watts, 2013). Each ‘construct of dichotomous oppositional difference’ is defined through the difference between the superior and inferior element (Hill Collins, 1986: S20). For example, objectivity is superior to and defined as lack of subjectivity; reason is the absence of inferior emotions, opinions, or instincts. The superior elements in the first column are entwined in their collective difference from the inferior elements in the second column. Thus the knower is an active agent whose objectivity stems from the distance maintained from that which is known, by virtue of being an outsider invested in reasoning and dispassionately looking at facts rather than steeping in their own values (Durkheim, 1894). The insider is inherently subjective, emotional, and unable to extract universal scientific claims due to their proximity to the subject matter; thus Durkheim (1894) warns sociologists against investigating personal matters. In other words, according to positivism, objectivity requires outsider status. Within positivism, ethics and politics are also assumed to be distinct from science while empirical scientific pursuits are construed as more legitimate forms of knowledge than non-empirical, philosophical reasoning which is deemed closer to values than to facts (Beauchamp, 2016; Knepper, 2001). Traditional knowledge, knowledge rooted in the body or emotions, and insider perspectives are also deemed inferior to outsider, empirical science (Harding, 1986; Spencer, 2015; Watts, 2013).

**Table 1.** Hierarchical dualisms in positivism.

Superior	Inferior
Knowing, doing	Being
Science	Religion, tradition, ethics, politics
Objective	Subjective
Outsider	Insider
Active knower	Passive known
Fact	Value
Mind	Body
Reason	Emotion, opinion, instinct
Universal	Local

These dualisms and especially the possibility of value-neutrality and complete objectivity within social and natural sciences have been contested especially by philosophers, historians, and sociologists of science such as Kuhn and Latour (Gingras, 2017; Rose, 1983; Rosenberg, 2018). Harding (1986) argues that *all* science is value-laden and tinted by social processes at all stages of inquiry: she demonstrates this through the cases of physics and mathematics, two disciplines which traditionally have been deemed the furthest removed from social influences. Instead, insisting on science’s so-called value-neutrality stems from a dogmatic belief that science is sacred and unique, which simply supports a mythologized understanding of science as progressive and obscures science’s failings, thereby shielding it from critique (Harding, 1986).

These limitations to positivism have been addressed in two ways. Feminist positivists have recognized that values do interfere everywhere in the research process which may lead to biases and even discriminatory scientific practices and results. However, such consequences are understood as manifestations of bad science which can be reduced by diversifying the demographics of scientists and by stricter adherence to positivist tenets (Harding, 1986; Rosenberg, 2018). Some have instead opted for constructivism which argues that truth is relative and socially constructed: the illusion of objectivity and the imperative to be objective are thus rejected in favor of embracing subjectivity (Berthelot, 2012; Haraway, 1988; Harding, 1986). However, constructivism is criticized as resulting in a lack of practical solutions to social problems: if everything is constructed, why bother attempting to change anything (Alcoff, 1988; Haraway, 1988; Harding, 1986). Feminist positivism and constructivism are considered poor solutions to the problems of positivism in that they still invest the same dualisms: objectivity and subjectivity are still construed as incompatible opposites (Alcoff, 1988; Haraway, 1988; Harding, 1986).

In sum, insiders cannot be objective per positivist epistemology as this status is framed as inherently subjective. One cannot be both the active knower and the passively known object of inquiry in positivism, thus another epistemology and conception of both subjectivity and objectivity is necessary.

### Standpoint epistemologies: subjectivity as the foundation for objectivity

Standpoint epistemologies, especially as formulated in Black and postmodern feminist thought, constitute some of the most influential critiques of positivism (Rosenberg, 2018). Standpoint epistemologies are rooted in a critique of positivist science’s self-affirmed cleavage from social and human processes; for example, Harding (1986) argues this so-called cleavage is anchored in a dogmatic mystification project. Black, Indigenous, and feminist scholars have shown how hierarchical dualisms in science have served to assert and maintain white, bourgeois, Western men’s interests both within science and society (Dixon, 1977; Haraway, 1988; Harding, 1986; Hill Collins, 1986; Mills, 1988; Watts, 2013). In positivism, white bourgeois Western men are assumed to be objective and scientific by default (Haraway, 1988). Androcentric bias in science, thus, does not result from a poor application of positivist principles but constitutes science-as-usual (Harding, 1986). Further, science has consistently been deployed to political ends: asserting the legitimacy of white, Western men’s dominance over non-white, non-Western women, people, and/or territories by insisting on a natural inferiority through proximity with emotions, bodies, traditions, nature, and the non-human (Dixon, 1977; Haraway, 1988; Harding, 1986). Behind myths of value-neutrality and progress, white Western science has, thus, been entwined with unethical eugenics and imperialist, colonialist, sexist, racist projects (Dixon, 1977; Haraway, 1988; Harding, 1986, 1992). Such consequences of hierarchical dualisms are summarized in Table 2.

**Table 2.** Consequences of hierarchical dualisms.

Superior/dominant	Inferior/subjugated
Man	Woman
White	Non-white
Western, Global North	Eastern, Global South
Culture	Nature
Civilized	Savage
Human	Non-human
Individualism	Communalism

In light of these critiques, standpoint theorists argue for a new epistemology acknowledging the legitimacy of different sources of knowledge and moving beyond hierarchical dualisms (Haraway, 1988; Harding, 1986; Rose, 1983). For instance, Harding (1986: 24) asks: ‘can we imagine what a scientific mode of knowledge-seeking would look like that was not concerned to distinguish between objectivity and subjectivity, reason and the emotions?’ Extending Marxist class-based analyses to gender, some feminists favor knowledge anchored in women’s standpoints in terms of lived and physical experiences (Rose, 1983). For instance, Rose (1983: 83) argues for a return to practicing natural sciences as a craft, as a caring ‘labor of love’ rather than an industrialized process. This would allow for a unity of manual, emotional, and mental labor evocative of women’s activities in general.

Black scholars and postmodern feminists recognize there is no single oppressed group and that identities may intersect: therefore they advocate for centering subjugated people’s knowledge in general rather than exclusively women’s knowledge (Haraway, 1988; Harding, 1986; Hill Collins, 1986; hooks, 2014). Subjugated people are deemed more aware of complex social processes and capable of an oppositional gaze, that is of ‘look[ing] from a location that disrupt[s]’ by virtue of experiencing the brunt of power dynamics which are invisible to oppressors (Du Bois, 1903; Haraway, 1988; hooks, 1992: 123; Mills, 1988). Victims of racism, sexism, and classism have tangible, lived experience which more accurately reflects the realities of racism, sexism, and classism than abstract conceptualizations formulated by those who have no lived experience (Mills, 1988).

This capacity to see social processes impacting subjugated people more clearly is theorized by and about Black people as ‘double-consciousness, this sense of always looking at one’s self through the eyes of others, of measuring one’s soul by the tape of a world that looks on in amused contempt and pity’ (Du Bois, 1903). Thus, bell hooks^2^ (2014: xvii) speaks of living on the ‘other side of the tracks’ as a Black youth in a Kentucky town: ‘We looked both from the outside in and from the inside out. We focused our attention on the center as well as on the margin. We understood both’. Hill Collins (1986: S14) refers to this perspective as that of the ‘outsider within’. She discusses Black women’s insights into the realities of living as Black *and* as white, gained through domestic and care labor performed in white suburban homes by those who never belong. This is compared to the standpoint Black women inhabit in academia. Indeed, Black women intellectuals have consistently and creatively drawn on their experiences of marginalization to call attention to the intersection of racism, sexism, and classism and to formulate ‘a special standpoint on self, family, and society’ (Hill Collins, 1986: S14). She, thus, encourages scholars to tap into their experiences ‘as a valid source of knowledge for critiquing sociological facts and theories’, adding that ‘sociological thought offers new ways of seeing that experienced reality’ (Hill Collins, 1986: S29–S30).

Building on Black feminist insights, Haraway (1988) redefines objectivity as rooted in subjective, situated knowledge. This feminist and situated objectivity is a reaction to the disembodied, unmarked, distant, and ethically irresponsible Western white man scientist’s gaze, which serves the status quo by objectifying and separating the knowing subject from their knowledge to extract universal truths. For Haraway, one can only be objective by acknowledging the partiality of one’s perspective, seeing through subjugated people’s eyes and from an identified location or standpoint. In other words, one can only be objective by proximity with and seeing from the insider’s perspective. This nuanced and localized rather than universal scope does not cleave the knower from what is known, which allows for caring, accountable, ethical, and politically engaged feminist science that avoids the impasse of constructivism (Haraway, 1988). This redefined objectivity can also be referred to as standpoint objectivity or strong objectivity (Harding, 1992, 2009).

Of note, the authors discussed in this section do not mobilize the insider and outsider concepts in the same ways as previously discussed. One must either be an insider or ‘do the necessary work to gain a rich and nuanced understanding of what such life-worlds are like, in order to think within that group’s standpoint’ according to Harding (1986, 2009: 194). Similarly, as long as one is not attempting disembodied observation, anyone can achieve objective feminist insights according to Haraway (1988). However, Haraway (1988) warns against the difficulty of attempting objectivity when looking inwards at oneself. This contrasts with Hill Collins’ (1986) invitation to creatively draw on one’s life experiences as an outsider within academia. According to hooks (2014), the key to effective feminist theorizing and movement-building is to center the most marginalized—namely, working class Black women—rather than exclude and talk over these groups.

Per standpoint epistemologies, an insider can be objective not despite, but through subjective experiences if they are open to their research subjects’ perspectives and their own. What does this entail for victimologists who have experienced victimization?

## Subjective insiders: victimologists who experience victimization

In this section, I present the case of victimologists who can be considered subjective insiders as they have lived experiences of victimization. To do so I define victimization to acknowledge the many ways victimologists can have such firsthand experiences. I then discuss advantages and disadvantages of insider research within victimology specifically.

### Victimization and victimologists

Within the context of standpoint theory, victim-related vocabulary has been used to refer to victims of oppression, a synonym for subjugated people (e.g. Harding, 1986; hooks, 2014). Victimization and oppression can indeed go hand in hand. In Canada, for instance, sexual orientation minorities, Indigenous people, low income people, and people who consume large amounts of drugs and alcohol face higher risks of violent victimization (Wemmers, 2017). Victimization can also be deployed as a marginalization or oppression tactic: think of hate crimes specifically targeting oppressed groups, war crimes, and victimization in genocidal and colonial contexts (Denov et al., 2020). However, victimization is not interchangeable with oppression within the context of victimology.

Definitions of victimization have traditionally relied on legalist definitions of crime (Fattah, 2010). However, as demonstrated within victimology, the pervasive emotional, psychological, physical, social, and financial consequences of victimization extend beyond the person who directly experiences an unlawful act (Wemmers, 2017). Thus, Wemmers (2017) presents a victim-centered typology which relies on ‘emotional or psychological proximity to the victimization’ (p. 60). As the targets of the unlawful act, *direct victims* have the closest level of proximity and have traditionally been recognized as victims. They may experience a range of short and long-term consequences including fear, depression, acute stress disorder (ASD), and post-traumatic stress disorder (PTSD). *Indirect victims* sit at the second level of proximity. They suffer from the consequences of the victimization through their close relationship with the direct victim. Through this proximity, they may experience helplessness, fear, complicated grief, and PTSD.

*Secondary victims* include witnesses of the unlawful act and bystanders (Wemmers, 2017). This group may also experience ASD and PTSD. Secondary victims include professionals who ‘have suffered harm in intervening to assist victims in distress’, such as first responders and care providers who are ‘repeatedly exposed to traumatic situations’ (Wemmers, 2017: 60). This latter category is likely to experience consequences such as vicarious traumatization, secondary trauma or compassion fatigue. Community members can be *tertiary victims* as certain forms of victimization may be part of a group’s shared history and instill fear in all community members (Wemmers, 2017). For example, even community members who have never directly experienced nor witnessed hate crimes, racial victimization, or systemic victimization can move through the world as tertiary victims, with the knowledge they could very well be victimized. Importantly, the above categories are neither mutually exclusive as one person may experience one or multiple victimization events, which can fit within one or multiple categories, nor are they static as someone who has never been victimized can be in the future.

The victim-centered typology is relevant to the current paper because like any other human being, victimologists can have direct, indirect, secondary, and/or tertiary experiences of victimization. For example, one author studying rape referred to herself as having an ‘investigator-victim perspective’ as she herself had been raped (Winkler, 1991: 14; Winkler and Wininger, 1994: 250) and another speaks of ‘survivor-anthropologists’ such as herself (Mahmood, 2008: 10). Some researchers of sexual assault, domestic violence, sexual harassment, and crimes targeting tourists explicitly identified themselves as direct victims of these unlawful acts (Brison, 2002; Cohen, 2019; Fletcher, 2018; Hayes and Jeffries, 2016; Kumar, 2017; Mackie, 2009; Mahmood, 2008; Minge, 2007; Railsback, 2020; Ross, 2020; Stanko, 1992; Winkler, 1991; Winkler and Hancke, 1995; Winkler and Wininger, 1994). Researchers studying their own marginalized communities also described experiences akin to direct, indirect, secondary, and tertiary transphobic and Islamophobic victimization (Gilliam and Swanson, 2020; Pearce, 2020; Zempi and Awan, 2017).

In other words, researchers who study victimization and themselves have certain experiences of victimization can be considered insiders. Keeping in mind the various degrees of proximity to victimization will prove important in the following sections.

### Advantages and disadvantages of insider status among victimologists

Victimization is inherently subjective: it ‘is not an objective reality but is rather a personal, subjective and essentially relative experience’ (Fattah, 2010: 50). Thus, insider victimologists both juggle with a subjective experience of victimization and a subjective relationship to victimological research itself. Subjective, insider victimologists face a series of advantages and disadvantages which are extensions of the insider-outsider debate discussed above.

Scholars looking inwards and analyzing their own experiences of rape and domestic violence through autoethnographic research have made important conceptual, methodological, and philosophical contributions to victimology through their insider status. Namely, victimologists have highlighted the importance of their insider status to fully represent the complex meanings of sexual and domestic violence (Brison, 2002; Hayes and Jeffries, 2016; Minge, 2007; Ross, 2020; Winkler and Wininger, 1994). Direct victims have tended to be silenced and excluded from scientific understandings of rape and trauma, which have traditionally centered perpetrators and stemmed from researchers who had never been raped (Brison, 2002; Campbell, 2002; Winkler and Wininger, 1994). Thus, outsider definitions of rape and its consequences have been sanitized to such a degree as to become meaningless to victims, whose own definitions rely less on legal terms and more on emotional consequences (Campbell, 2002; Winkler, 1991; Winkler and Hancke, 1995; Winkler and Wininger, 1994). Winkler (1991) redefines rape as social murder whereas Hayes and Jeffries (2016) reframe domestic violence tactics as romantic terrorism. Brison (2002) presents philosophical implications of sexual assault, whereas Montmagny Grenier (2021) identifies key limits to reflexivity when researchers are assaulted by key informants and negate how their bodies are eroticized.

Insider status can be advantageous to victimologists and their research participants. Indeed, being an insider can help identify topics omitted by outsiders; formulate more sensitive questions which increase participants’ openness; develop more empowering methodologies in which participants are co-investigators rather than mere informants; be more effective in data analysis; and benefit from deeper understanding of their subject matter (Stoler, 2002; Winkler and Wininger, 1994; Zempi, 2017; Zempi and Awan, 2017). Some researchers who have directly been victimized also identify personal benefits to their research: finding hope, voice, and identity; fighting stigma and shame; recognizing resilience; surviving and healing (Fletcher, 2018; Kumar, 2017; Mackie, 2009; Mahmood, 2008; Railsback, 2020; Stoler, 2002). In that sense, insider victimology can help victims: those who conduct research and those who do not.

However, conducting victimological research as an insider is not without its risks. Researchers who are direct or secondary victims report emotional, psychological, and physical tolls such as frustration, sadness, pain, withdrawal, isolation, guilt, suicidal thoughts, loss, fear, anger, powerlessness and trauma as well as symptoms of compassion fatigue and secondary trauma (Campbell, 2002; Coles et al., 2014; Gilliam and Swanson, 2020; Kinard, 1996; Pearce, 2020; Spencer, 2016; Williamson et al., 2020; Zempi, 2017; Zempi and Awan, 2017). One article is framed as a cautionary tale regarding ‘unexpectedly traumatic’ insider research as despite its advantages, this type of research can weigh especially heavy on graduate students and members of marginalized communities (Gilliam and Swanson, 2020: 903). Indeed, navigating victimization and its consequences on oneself, one’s loved ones, one’s community, and one’s research participants all at once can imperil the insider victimologist. For instance during her PhD, which involved distressing research into issues she also faced as a member of the same marginalized community, Pearce (2020) became her friend and roommate’s caregiver, then found her after she committed suicide; she was also supporting friends who had experienced sexual assault and working through her own direct victimization. She speaks to marginalized researchers’ survival of oppression and traumatic fieldwork, arguing that ‘if the researcher cannot survive the research process, then something has gone horribly wrong with the methodological design of their study’ (Pearce, 2020: 3).

Finally, agency may be an ambiguous issue for insider victimologists. Contrary to other researchers, victimologists who have been victimized may not benefit from the luxury of choosing to investigate unlawful acts they never chose to experience. However, ‘it is in that lack of choice that a crime victim fully comprehends the status of that subject matter’ (Winkler and Wininger, 1994: 251). Zempi and Awan (2017) describe their choice to conduct dangerous autoethnographic fieldwork and gain the advantages of insiders. However, they ‘do not argue that one needs to turn oneself into a victim and to experience victimhood, in order to write about it’; the authors ‘acknowledge that this would be both analytically and ethically problematic’ (Zempi and Awan, 2017: 15). The ambiguity between advantages and disadvantages of insider victimological research sets the table for deconstructing dualisms related to insider and outsider status.

## Deconstructing dualisms among victimologists

In this section, I deconstruct insider–outsider and subjectivity–objectivity dualisms as they pertain to victimologists then conclude on the untenability of positivism in victimology.

### Complicating insider and outsider status in victimology

Many authors have contested the possibility of fully being an insider or an outsider. Indeed, even when researchers share one identity with participants, groups are heterogeneous: researchers never share the exact same sociodemographic characteristics and life experiences as their participants and thus are more likely to be partial insiders than complete insiders. Although a researcher may perceive themself or be perceived by researchers as an insider by virtue of one shared characteristic, participants may view them as an outsider due to the high education level inherent to being an academic (Serrant-Green, 2002). A researcher may simultaneously be an insider and an outsider, relating to participants—or not—based on race, gender, class, sexuality, disability, religion, migration status, education and employment, and more (Acker, 2001; Dwyer and Buckle, 2009; Jewkes, 2012; Labelle, 2020; Pollack and Eldridge, 2015; Serrant-Green, 2002; Stockdale, 2017; Zempi, 2016, 2017; Zempi and Awan, 2017). Discussing research on prisons and prisoners, Pollack and Eldridge (2015: 138) argue in favor of ‘disrupting the divide’ between insider and outsider research through choice of theoretical framework and methodological choices in which data collection, analysis, and dissemination involve and serve the most marginalized.

The insider–outsider binary is further complicated as pertains to victimology because victimization is not static thus neither insider nor outsider status can be static. Some victimologists can be victimized and acquire insider status over their lifetimes. Indeed some researchers have experienced direct victimization during the course of their graduate studies or fieldwork (Coles et al., 2014; Gilliam and Swanson, 2020; Mahmood, 2008; Montmagny Grenier, 2021; Stanko, 1992). At their participants’ urging, one Muslim and one non-Muslim researcher donned visibly Muslim clothing for 1 month to expose themselves to Islamophobic victimization, showing it is possible to become insiders (Zempi, 2017; Zempi and Awan, 2017).

Further, victimologists who have experienced victimization may technically be considered insiders, but their experiences can differ significantly from those of participants who experience a different type of unlawful act. For instance, a direct victim of burglary researching direct victims of human trafficking may not have very much common ground and remain an outsider. Victimologists’ relationships to their victimizations may also change over the course of their lifetime and affect insider or outsider status. Like Stoler (2002), victimologists may believe they are outsiders due to suppressed memories of victimization which then re-emerge over the course of their research. Victimologists may also integrate their experience of victimization and heal, thus engage in insider victimology without the disadvantages discussed above. Finally, an expansive victim-centered typology entails an equally expansive understanding of insider status, beyond direct victims studying direct victims. Indeed, victimologists with different degrees of proximity to the same type of unlawful act as their participants may be uniquely situated insiders: for instance, researchers who grew up in a household where one parent abused the other (indirect victims) may be quite sensitive to direct victims’ experiences.

Perhaps due to the stigma of victimization and victimization myths according to which victimized people are ‘others’, ‘them’ rather than also ‘us’, ‘we’ (Campbell, 2002), there is a shortage of literature where researchers name their position as victims or survivors of crime (Brison, 2002; Ross, 2020). This might also be because despite the expansive victim-centered typology, a researcher who exclusively experienced indirect, secondary, or tertiary victimization may not consider themselves an insider. Consequently, insider and outsider status are further complicated in victimology due to self-identification as a victim or survivor of an unlawful act. Some clinician-researchers could be considered secondary victims due to vicarious traumatization stemming from repeated and long-term exposure to direct victims’ horrific stories in their clinical practice, yet none whose work I read concluded this nor considered themselves insiders.

Through repeated and long-term exposure to participants’ stories of victimization, victimologists may also *become* insiders by physically and emotionally embodying their research participants’ trauma. An emerging body of literature identifies symptoms of vicarious traumatization and secondary traumatic stress as well as compassion fatigue among researchers investigating victimization and other emotionally sensitive topics (Campbell, 2002; Coles et al., 2014; Coles and Mudaly, 2010; Dickson-Swift et al., 2009; Etherington, 2007; Nikischer, 2019; Stoler, 2002; Williamson et al., 2020). Indeed ‘victim-related research is a thoroughly traumatic endeavor’ (Kinard, 1996; Spencer, 2016: 118). This is especially the case in light of researchers’ empathy and sensitivity to participants’ distress as well as frequent, cumulative exposure to participants’ trauma at all stages of the research process, and may be exacerbated for vulnerable researchers including those who have experienced direct or indirect victimization (Coles et al., 2014; Coles and Mudaly, 2010). The emotional detachment that is presumably essential to scientific endeavors and lack of a clear helper role can also contribute to researchers’ distress (Campbell, 2002; Coles and Mudaly, 2010; Dickson-Swift et al., 2009; Mulla, 2014). Whereas care professionals draw on compassion as a resource and have the satisfaction of assisting victims, researchers are socialized to attempt objectivity, manage and hide their emotions and to extract the victim’s story without necessarily giving back, feeding into feelings of helplessness and isolation (Campbell, 2002; Coles and Mudaly, 2010; Dickson-Swift et al., 2009; Mulla, 2014). This may be especially difficult to clinician-researchers: Coles and Mudaly (2010: 61) discuss how their clinical tasks shielded them emotionally as they ‘limited client/patient stories and emotions to those relevant to the professional task’, which contrasted with the research interview where they ‘encouraged participants to tell their stories in as much detail as possible’.

Qualitative, interview-based victimological research can be especially emotionally challenging and even traumatizing to researchers as the empathy which make such interviews safer for participants may be detrimental to researchers’ well-being (Coles, 2004). Kinard (1996) also discusses the emotional consequences research assistants reported in reviewing case records of child maltreatment. Etherington (2007: 91) discusses her interview transcriber’s distress in being an ‘outsider witness’ to adult survivors’ stories of childhood trauma, noting the latter did not experience vicarious traumatization, while Woodby et al. (2011) discuss the unique, cumulative emotional stress of coding sensitive data. Stoler (2002) finds transcribing and analyzing data to be more distressing than responding to emotional content while conducting interviews, for which she is well trained as a clinician. Despite recognizing these potential impacts on researchers and research teams, no author self-identifies as gaining insider status through vicarious victimization.

Victimologists’ insider and outsider status is further complicated by a study’s methodological design. Winkler referred to her research participants as investigator-informant-victims as they were involved in co-constructing and analyzing the data (Winkler and Wininger, 1994) while Kumar (2017) refers to her participants as coresearchers. In these cases, authors were insiders who shared similar experiences of direct victimization with their participants. In one participatory research studying Rwandan youths’ experiences as secondary victims of genocidal rape, three youths born of genocidal rape received research training and were involved at all stages from design to data collection to co-authoring (Denov et al., 2020). Is this insider research, or must every research team member have firsthand experience of victimization? The latter criterion would change little for autoethnography but would imply bigger research teams could almost never do insider victimology.

### Implications for victimologists’ subjectivity and objectivity

Complicating the insider–outsider binary within victimology has important implications for victimologists’ subjectivity and objectivity. Many autoethnographers studying gender-based violence discuss what their insider status and research method imply for subjectivity and objectivity (Brison, 2002; Fletcher, 2018; Hayes and Jeffries, 2016; Mahmood, 2008; Montmagny Grenier, 2021; Nikischer, 2019; Pearce, 2020; Ross, 2020; Winkler and Hancke, 1995; Zempi, 2016). Indeed, doing insider research and drawing on one’s life experiences tends to be discredited as inherently subjective, narcissistic, self-indulgent, lazy ‘*me*search’ (Jewkes, 2012; Zempi, 2017).

Yet all researchers can fall into the ‘space between’ insider and outsider status, never fully one nor the other, or constantly moving between these porous categories (Acker, 2001; Dwyer and Buckle, 2009). Relying on the positivist definition of objectivity, according to which only outsiders can be objective, thus remains impossible in victimology: subjectivity will be present, one way or another. For instance, the will to ‘prevent crime and the hurt and pain criminals cause their victims’ (Knepper, 2001: 3) can be an important motivation for becoming a victimologist: lifetime direct, indirect, and tertiary victimization significantly influence diverse urban college students’ motivations for studying criminal justice (Eren et al., 2019). Students who experienced victimization expressed interest in social justice in the sense they strongly showed ‘a desire to protect people from oppression’, ‘make a difference in the community’ and ‘be treated equally’ (Eren et al., 2019: 522).

Even a victimologist who appears to be a full outsider cannot maintain value-neutrality due to this field’s inherently emotional, moral, and political subject matter. By virtue of being humans, living in a social context, and carrying their own grief and trauma, all victimologists can have strong opinions and feelings about victimization. This field has a strong affective and cognitive component, as evidenced by studies of fear of victimization (Jewkes, 2012), as well as researchers’ overall experiences of studying childhood sexual abuse (Stoler, 2002). As Mulla (2014: 232) puts it, ‘when one works on sexual assault, one simply expects to be emotionally taxed’; the same could be said for researching many forms of victimization including crimes against humanity. Novice outsider researchers may also experience emotions such as fear and anxiety before starting fieldwork (Jewkes, 2012). Regardless of whether they or their loved ones have such experiences, victimologists may consider certain unlawful acts to be inherently immoral, for instance defrauding the elderly. Through their research, victimologists may experience fear of victimization targeting themselves, their loved ones, or their community (Coles and Mudaly, 2010).

Thus, Fattah’s (2010) dispassionate, objective, outsider victimology strikes me as an unattainable ideal considering this field’s very subject matter. When researching sensitive topics which one may fear experiencing, ‘accounts of research . . . that rely on rhetorics of objectivity or distance might be both unhelpful to researchers and misleading for research audiences’ (Johnson and Clarke, 2003: 423–424). In other words, positivism is untenable in victimology.

## Embracing subjectivity as the foundation for objectivity in victimology

If insider and outsider status are complicated and all victimologists can be subjective, the criteria for a positivist version of objectivity are untenable within this field. Indeed, positivism posits that objectivity requires outsider status and is diametrically opposed to insiders’ subjectivity. Because standpoint epistemologies recognize the impossibility of conducting research that is not value-laden and the possibility of insider research, I argue the solution to victimology’s problem lies therein. I propose to rely on Haraway’s (1988) version of objectivity in which one can be an insider or an outsider if one embraces subjectivity to see from the insider’s perspective and from an identified standpoint.

Although Haraway (1988) proposes a novel definition of objectivity in which subjectivity is the foundation, she does not delve into its day-to-day implementation. What does seeing from the insider’s perspective and from an identified standpoint entail in practice? Because research methodologies are extensions of epistemology (Dixon, 1977; Labelle, 2020), in this section, I present a practical guide for researchers who wish to embrace subjectivity as a foundation for objectivity. To do so I weave together methodological tools relevant to standpoint epistemologies with arguments and examples from more-or-less insider victimological research as reviewed above. These tools especially originate from intersectionality, a framework derived from standpoint epistemologies and Black feminism which considers entwined oppressive social structures to understand how multiply marginalized people—especially Black women—move through the world (Crenshaw, 1991; Hill Collins, 2019). Intersectionality is a critical social theory, methodology, and praxis that has made its way into criminology and victimology (Crenshaw, 1991; Hill Collins, 2019; Potter, 2015). This guide is based on components deemed diametrically opposed to objectivity and tied to subjectivity, according to positivists: location, emotion and body, and ethics (see also Labelle, 2020). Indeed, embracing subjectivity as a foundation for objectivity is an ongoing process which requires work and can take a variety of forms.

### Location

Seeing from an insider’s perspective can mean different things. One way is for researchers to build projects which start from the researched population’s daily wants, needs, and desires rather than topics prescribed as interesting by one’s home discipline (Harding, 2009). This can be achieved through familiarization with the population’s accounts of self appearing in media, art, essays, qualitative scholarship, and more (Clair, 2021). Within standpoint epistemologies, regular people are deemed capable of naming and even theorizing their own experiences (Clair, 2021; Hill Collins, 1986; hooks, 1992, 2014). Victim-centered approaches that focus on victims’ self-definitions and needs rather than on causes of crime and victimization (Fattah, 2010; Wemmers, 2017) are examples of scholarship starting from an insider’s perspectives.

To see from an insider’s perspective and from an identified standpoint, researchers need to center research subjects and attempt to see through their eyes through proximity rather than detachment. This process starts by the identification and critical examination of one’s own social location as a researcher in relation to the social location of the research population, whether one appears to be an insider or an outsider at first glance (Serrant-Green, 2002). Depending on the research topic, this can include reflecting on one’s settler status, race, gender, social class and level of education, sexuality, (dis)ability, religion, migratory status, experiences of victimization, and more. Although ‘the core ingredient is not insider or outsider status but an ability to be open, authentic, honest, deeply interested in the experience of one’s research participants, and committed to accurately and adequately representing their experience’ (Dwyer and Buckle, 2009: 59), delineating the researcher’s standpoint is important as it allows knowledge to be placed in the context in which it was produced. Local, admittedly partial knowledge rather than claims of universal truth are privileged ‘as an antidote to scholarship that, in the guise of universality, tends to silence those who most need to be heard’ such as rape victims (Brison, 2002: 6). Think of it as hanging a photograph of a landscape next to a map and diagram indicating the precise vantage point and angle from which it was snapped: the landscape looks beautiful from your perspective, but it might not be so for everyone nor from every angle. That is objectivity.

Recognizing the partiality of one’s knowledge, whether one is technically an insider or an outsider also allows researchers to commit to learning about their research subjects’ experiences and perspectives through self-discipline (Bartlett, 1990). By identifying one’s location and why they are drawn to a given topic, victimologists can identify their expectations and assumptions and attempt to minimize power dynamics (Harding and Norberg, 2005). Victimologists can also reflect on how wise it is to initiate a project and anticipate potential difficulties such as the resurfacing of traumatic memories, which happened to Stoler (2002) while researching childhood sexual abuse. I recommend that victimologists continuously ask themselves: ‘How similar am I to the population I wish to research/am researching? How different am I to this population? How might my own baggage be useful for or interfere with this research?’.

By asking these questions continuously, victimologists can identify and embrace commonalities with research participants to imagine what it is like in their shoes and make sense of results, while ensuring they do not mistake themselves for participants and misrepresent data. As discussed above, one’s own experiences can be useful to identify gaps in knowledge, develop an interview protocol, think of probes, and more. Yet when analyzing data, the material should be allowed to speak for itself. One’s differences can be used as a starting point to find and learn from this population’s insider knowledge, including through educational content on social media, artwork, academic and non-academic writing. Like the map and diagram next to the landscape photograph taken as an example above, I encourage victimologists to be transparent as to their vantage point throughout the project and when communicating their findings. This can be done formally through a positionality statement placed in a paper’s methodology section for instance, and informally by readily answering potential participants’ interrogations. Table 3 presents an example of my own positionality statement for this paper.

**Table 3.** Example of a positionality statement.

*I came to question the meanings of objectivity and subjectivity as well as outsider and insider status in victimology after conducting interviews on leisure in women’s prisons. As a white, non-binary trans PhD student who had never been incarcerated, I assumed I was an outsider. Yet many participants unexpectedly revealed their experiences of gender-based victimization in horrific detail, viscerally evoking my own experiences and reactivating my traumas. This painful proximity to participants prompted a personal and intellectual crisis which I set out to resolve in this paper.*

There is one caveat. Many activists have clamored that outsiders should not research oppressed groups given the historical tendency to deploy science for eugenics; to research themes that make no difference on a daily basis; to misrepresent oppressed groups’ interests; and to extract knowledge without giving back (e.g. Tagonist, 2009). I am not claiming outsiders should necessarily conduct victimological research pertaining to any oppressed group. For example, based on positivist epistemologies, white scholars have traditionally been assumed to be objective when studying matters affecting Black people due to outsider status and an epistemology serving white scholars’ interests (Serrant-Green, 2002). Based on standpoint epistemology, Black victimologists studying Black people’s experiences of racial profiling by police are uniquely situated to practice objectivity by virtue of seeing the whole picture and by embracing theirs and their participants’ subjectivity. White victimologists *could* also be objective by reflecting on positionality; whether white victimologists *should* do this type of research is another matter. The onus remains on victimologists to pay attention to what vocal members of marginalized groups have to say about research conducted by non-members, to respect these perspectives, and to abstain when necessary. Indeed embracing insiders’ subjectivity entails respecting its legitimacy as a valid source of knowledge, including when this leads to a heartfelt ‘no’. Objectivity can be achieved by embracing subjectivity, but oppressive or extractive dynamics cannot be wished away by erasing important structural differences between researcher and researched; thus, other factors such as emotions, bodies, and ethics come into play and are discussed in the following sections.

### Emotions and bodies

To see from the insider’s perspective, victimologists must not only recognize and embrace the importance of research participants’ subjectivity but also their own. Although subjectivity has often been discussed in terms of values, opinions, or political inclinations, emotions and bodies are also components of subjectivity which have traditionally been considered ‘the anathema to academic research’ as noted by many victimologists (Brison, 2002; Campbell, 2002; Dickson-Swift et al., 2009: 63; Jewkes, 2012; Mahmood, 2008; Montmagny Grenier, 2021; Spencer, 2015). Yet ‘theory must base itself on the initial premise that all persons, including the theorist, have a fleshy, material identity that will influence and pass judgment on all [knowledge] claims’ (Alcoff, 1988: 434; see also Bartlett, 1990). For example, after graphically describing violent sexual and physical assaults which necessarily affected her body, which she refers to as writing ‘from the bones’, Mahmood (2008: 11) argues in favor of ‘a spiritual, political, and intellectual paradigm which recognizes that, unspoken or not, values of the heart are as central to our field as those of the mind’.

Victimologists would benefit from embracing all components of subjectivity including emotions and embodied selves, which can be valuable sites of reflection and sources of knowledge throughout the research process as evidenced by autoethnographic victimological research (Brison, 2002; Campbell, 2002; Jewkes, 2012; Kanuha, 2000; Mahmood, 2008; Montmagny Grenier, 2021). For instance, failing to account for ways researchers’ bodies are subjectively gendered, sexualized, and sites of power dynamics can be physically dangerous during fieldwork and hinder critical reflections on fieldwork itself (Montmagny Grenier, 2021). Spencer (2015) notes victimology’s treatment of bodies as untrustworthy sites of knowledge despite the ‘primacy of bodies to victimization’ (p. 32). Centering bodies could help victimologists make sense of victimized individuals’ trauma and pain in a less abstract manner, while helping to account for ‘ways in which the researcher’s body impacts the act of conducting victimological research’ throughout data collection, analysis, and writing (Spencer, 2015: 40). A process wherein the researcher is a ‘body-subject examining victimized body-subjects’ therefore ‘effaces the object and subject divide within victimological inquiry’ (Spencer, 2015: 41).

Victimological research can take a tremendous emotional toll and involve emotion work by participants and emotional labor by researchers; both should be recognized and addressed throughout (Campbell, 2002; Kinard, 1996; Spencer, 2016). Emotion work refers to the management of one’s feelings and expressions in the private space, whereas emotional labor refers to this management when it is expected in professional spaces such as customer service (Hochschild, 1979, 2012). Rehashing traumatic experiences can help research participants externalize negative emotions (Campbell, 2002). However, this can also place them in a position where they need to perform significant emotion work as they are asked to ‘relive painful experiences and feel unwanted emotions’ which are not necessarily acknowledged nor compensated (Labelle, 2020: 14). For example, one participant spun into an emotional crisis during and after the interview to such an extent that Labelle (2020) needed to revisit her ethical commitment to this population and ultimately chose to reorient her doctoral project. As for academics, Dickson-Swift et al. (2009) found that researching sensitive topics especially through qualitative methods is emotion work, meaning academics engage in significant labor related to showing, embodying, and constantly managing their emotions throughout the research process while also navigating dilemmas as to the place of emotions in academia. Discussing their approach as insiders who experienced Islamophobic victimization, Zempi and Awan (2017: 15) underscore the ‘emotional labour that is required to make the connections between experiences and insight/knowledge’.

The emotional labor put into understanding insiders’ experiences can, however, be enriching. Rather than simply *thinking* of rape, Campbell (2002) and her research team gained more significant insights when they opened themselves to *feeling* rape victims’ emotions and becoming emotionally involved. Indeed, although the team thought they were well prepared, data collection challenged their core assumptions. Team members realized that as women, they could easily have been 1 of the 100 rape victim participants, and none were immune from experiencing rape in the future. Feeling loss, pain, fear, anger, and hope led to a shift in the team’s position, ‘creating new vantage points—everything looked different from where we now stood’ (Campbell, 2002: 107). Through emotional proximity with participants, the team was able to identify and theorize an important gap in the literature: they found that classical and legal definitions of rape are so sanitized they become meaningless to victims. Rather, rape is defined by victims in deeply emotional terms. The author concludes that emotions can be an intellectual resource in research:

The emotional pain of rape is not a threat to ‘objectivity’, nor is it necessarily a threat to the well-being of the researcher. Emotions can be an important resource for science, and the emotionality of rape is essential to its understanding. (Campbell, 2002: 122)

Reflexivity is an important avenue in gaining intellectual insights from emotions (Jewkes, 2012) and more widely in embracing subjectivity as foundation for objectivity. Although this practice can be defined in many ways, at its core reflexivity involves ‘complex relationships between how we know, what we know and who we are’ (Etherington, 2004: 46). Researchers practice reflexivity when they critically examine their social and cultural location, embodied emotions, values, politics, and more; self-awareness, transparency, and rigor are key. As Etherington (2004) notes, ‘reflexivity is not the same as subjectivity but rather it opens up a space between subjectivity and objectivity where the distinction between content and process becomes blurred’ (p. 47). Reflexivity can be practiced by keeping a ‘journal of thoughts, feelings, and behaviours’ which help process emotions, self-examine, and note insights throughout a research project (Stoler, 2002: 271). Reflexive personal writing can reduce relations of power between researcher and researched—which is especially crucial while working with sensitive topics as is often the case in victimology—while also helping tackle the ethical issues surrounding such power dynamics (Campbell, 2002; Etherington, 2004; Montmagny Grenier, 2021). The positionality statement in Table 3 is an example of reflexivity. Other avenues include asking ‘why am I reacting this way to this information?’, consciously detailing why one chooses to take the project in a certain direction, and critically interrogating whether one’s interpretation of data does justice to participants’ experiences.

Although questions of location, emotion, body, and reflexivity have mostly been addressed within the context of qualitative research, this does not mean embracing subjectivity to achieve objectivity is limited to certain methodologies. Reflexivity and questioning one’s positionality can always be deployed including in quantitative research. Indeed, the researcher’s subjectivity will also appear throughout quantitative research, from inception to methodological design to analysis and research mobilization (Harding, 1986).

### Ethics

In victimological research, embracing subjectivity and emotions requires an ongoing commitment to ethics. Indeed Spencer (2016) argues that for victimologists ‘to sit back in their Archimedean ivory tower armchairs and fail to respond to the pain and anguish of their research participants is . . . as ‘morally indefensible’ as the natural scientist that sacrifices rabbits in the name of producing a tear free eye make-up remover’ (p. 110). Drawing on their experience researching unhoused individuals, the author outlines what ethics should be in victimology: an ethics of bearing witness to oneself within the experience, to others being victimized, and to the process of witnessing. Others discuss the importance of bearing witness to victimization as well as an ethic of caring in emotionally demanding research (Brison, 2002; Campbell, 2002; Gilliam and Swanson, 2020). This ethic of caring involves a mutually beneficial ‘emotional connection and concern’ for the participants, for what becomes of the research, as well as for oneself and the research team (Campbell, 2002: 127). An ethical commitment to social justice and toward working to improve the lives of subjugated groups has been central to standpoint epistemologies from the get-go (Crenshaw, 1991; Harding, 2009; Hill Collins, 2019; Hill Collins and Bilge, 2016; hooks, 2014).

Embracing subjectivity, especially emotional and embodied components, must be done with utmost care to protect the victimologist’s wellbeing. Victimologists who choose to embrace subjectivity in their pursuit of objectivity can embody care ethics by engaging in self-care strategies throughout a research project. This includes being aware of and prepared to respond to participants’ emotions; relying on psychotherapy and peer support; engaging in creative, spiritual, and rewarding activities; using fieldnotes and reflexive journals; limiting exposure to traumatic content through a manageable workload; ending the work day with a ritual; and ensuring the research has positive outcomes (Coles, 2004; Coles and Mudaly, 2010; Dickson-Swift et al., 2009; Johnson, 2009; Kinard, 1996; Kumar, 2017; Kumar and Cavallaro, 2018; Nikischer, 2019; Stoler, 2002). Being prepared may be possible when researching sensitive issues such as violence and abuse or when researching experiences akin to one’s own. However, researchers may experience traumatic events or unexpectedly sensitive issues during the research process itself, thus various and ongoing types of self-care are crucial for researcher well-being (Kumar and Cavallaro, 2018).

An ongoing commitment to ethics goes beyond the individual victimologist: institutional, organizational, and cultural responsibilities in caring for researchers beyond risk-based, bureaucratic requirements are also important (Spencer, 2016). For example, prioritizing safety strategies for researchers within institutional review board practices; offering better training to novice researchers within graduate curricula; supervising committees and departments mentoring and supporting students adequately; encouraging administrative flexibility to delays necessary in processing emotional pain; funding counseling within research grants; valuing collaborative rather than isolated, individual research; changing academic structure and culture (Bloor et al., 2010; Coles and Mudaly, 2010; Gilliam and Swanson, 2020; Kinard, 1996; Kumar and Cavallaro, 2018; Nikischer, 2019; Pearce, 2020; Stanko, 1992).

## Discussion

Any researcher can have experienced victimization or experience victimization in the future thus Fattah’s (2010) take on good, detached, objective victimological science leaves the field at an impasse which I attempted to resolve throughout this paper. I have asked: what are the epistemological and practical implications of research conducted by researchers who have firsthand experiences of victimization? What lessons can be retained by other victimologists and researchers in general? How can these epistemological considerations be applied in practice? I have examined the meanings of insider and outsider status and the implications for objectivity and subjectivity per positivist and standpoint epistemologies. I presented the case of victimologists who have been victimized as well as advantages and disadvantages of this form of insider research. I deconstructed insider–outsider, subjectivity–objectivity dualisms as pertains to victimologists, concluding that all victimologists can be subjective whether they are technically insiders or not. Drawing on examples from victimology, I discussed how all researchers can embrace their own and their participants’ subjectivity as a resource for objectivity by examining location, emotions and bodies, and ethics throughout the research process. To summarize my argument: victimology can be partisan, passionate, subjective, *and* constitute good science based on standpoint epistemology.

The ‘basic tenets in a given field have been formulated by people, who, in turn, are shaped by their environment’ (Dixon, 1977: 120). Standpoint theory attempts to reform science and disciplinary conventions by challenging such core assumptions (Harding, 2009). In a way this is what Fattah (2010) argues for as he addresses the tendency of aligning victimological research with the victim’s rights movement, which amplifies the concerns of a subset of victims and is instrumentalized to justify more punitive institutions. For instance, the concerns of victims of gender-based violence like rape are constantly represented whereas victims of state violence are nowhere to be seen (Fattah, 2010). The rights and needs of certain victims are then pitted against those of offenders. This makes no sense to Fattah (2010) because in practice the same disenfranchised groups tend to be both victimized and criminalized; think of drug users and sex workers. Ultimately, Fattah favors a shift toward restorative rather than punitive justice and argues for a criminology/victimology which would cease to be complicit in abusing the powerless (McCormick, 2021).

Although I have framed this paper as a challenge to Fattah’s (2010) assertions, his intentions and mine are not incompatible. Standpoint epistemology is all about centering the voices of marginalized and vulnerable individuals: this necessarily includes victims who have been deemed unworthy of attention and socially expendable. Through his political engagement, Fattah himself has acted in ways that are more compatible with standpoint than positivist epistemologies. Indeed Fattah ‘was one of many leading the successful fight in Canada against the death penalty’ (Canadian Heirloom Series, 2003) and in a recent book he encourages criminologists to side with justice and denounce abuses of power (McCormick, 2021). Whereas political engagement and the fight for social justice are unscientific pursuits within positivism, they are inherent to standpoint epistemologies (Harding, 2009).

As victimologists, we can all have experienced victimization and even when we have not, centering the subjectivities of those who have—insiders—can enrich our research and make it more objective by exposing aspects that have remained unseen. This brings us back to the place of subjectivity in research in general. As social scientists, we are concerned by the social phenomena we study; pretending otherwise is tunnel vision. Roughly put, sociologists are members of society. Psychologists have psyches. Political scientists have political stances and experience the effects of politics. We are all lab rats working from inside the social experiment and we need to grapple with this reality to produce better science. If victimology can draw on subjectivity and insider status as a resource for objectivity, other disciplines and fields can attempt this shift as well.

Although Descartes’ (1637) work inspired hundreds of years of positivism, he did not believe in prescribing certain ways of thinking nor of doing science, especially not in the name of tradition. In closing, I am not prescribing that all victimologists should adopt subjectivity as objectivity, nor that standpoint and positivist epistemologies are the only two options. I am simply presenting an alternative manner of envisioning research beyond the tradition of positivism. This, I hope, speaks to some fellow victimologists and contributes to increased recognition of the presence, voices, and the legitimacy of subjectivity and insider perspectives within victimology and academia at large (see Brison, 2002).

## Notes

1. This terminology stems from a ‘social and political life [which] saw race, gender, and sex as white, male, and heterosexual; academia and science reflected that same homogeneous state; and lands of the non-Western world were occupied by the objects of science commonly known as “natives” and “savages”’ (Kanuha, 2000).

2. bell hooks famously does not capitalize her name so focus is placed on her ideas rather than her, and to distinguish between herself and her grandmother from whom her pen name is inspired.
